# MZe786, a hydrogen sulfide-releasing aspirin prevents preeclampsia in heme oxygenase-1 haplodeficient pregnancy under high soluble flt-1 environment

**DOI:** 10.1016/j.redox.2020.101768

**Published:** 2020-10-24

**Authors:** Homira Rezai, Shakil Ahmad, Faisal A. Alzahrani, Lissette Sanchez-Aranguren, Irundika HK. Dias, Swati Agrawal, Anna Sparatore, Keqing Wang, Asif Ahmed

**Affiliations:** a Mirzyme Therapeutics, Innovation Birmingham Campus, Faraday Wharf, Holt Street, Birmingham, B7 4BB, United Kingdom; b Aston Medical Research Institute, Aston Medical School, Birmingham, United Kingdom; c Department of Biochemistry, ESC Research Unit, Faculty of Science, King Fahd Medical Research Center, King Abdulaziz University, Jeddah, 21589, Saudi Arabia; d Department of Maternal Fetal Medicine, Mt Sinai Hospital, University of Toronto, Toronto, Canada; e Department of Pharmaceutical Sciences, University of Milan, Milan, Italy; f President's Office, University of Southampton, University Road, Southampton, SO17 1BJ, UK

**Keywords:** Preeclampsia, Hydrogen sulfide, Heme oxygenase-1, sFlt-1, Hypertension

## Abstract

Preeclampsia affects one in twelve of the 130 million pregnancies a year. The lack of an effective therapeutic to prevent or treat it is responsible for an annual global cost burden of 100 billion US dollars. Preeclampsia also affects these women later in life as it is a recognised risk factor for cardiovascular disease, stroke and vascular dementia. Our laboratory demonstrated that preeclampsia is associated with high soluble fms-like tyrosine kinase 1 (sFlt-1) and low heme oxygenase-1 (HO1/Hmox1) expression. Here we sought to determine the therapeutic value of a novel H_2_S-releasing aspirin (MZe786) in HO-1 haploid deficient (Hmox1^+/−^) pregnant mice in a high sFlt-1 environment. Pregnant Hmox1^+/−^ mice were injected with adenovirus encoding sFlt-1 or control virus at gestation day E11.5. Subsequently, Hmox1^+/−^ dams were treated daily with a number of treatment regimens until E17.5, when maternal and fetal outcomes were assessed. Here we show that HO-1 compromised mice in a high sFlt-1 environment during pregnancy exhibit severe preeclampsia signs and a reduction in antioxidant genes. MZe786 ameliorates preeclampsia by reducing hypertension and renal damage possibly by stimulating antioxidant genes. MZe786 also improved fetal outcome in comparison with aspirin alone and appears to be a better therapeutic agent at preventing preeclampsia than aspirin alone.

## Highlights

• Partial loss of heme oxygenase-1 under high soluble Flt-1 causes severe preeclampsia compared to high sFlt-1 alone.
• MZe786, hydrogen sulfide releasing aspirin prevents preeclampsia by suppressing maternal hypertension and kidney injury.
• MZe786 is able to rescue pregnancy and improves fetal outcome despite the persistent high levels of sFlt-1.
• MZe786 is a superior therapeutic candidate than aspirin in preventing preeclampsia.

## Graphical abstract

**Figure:**
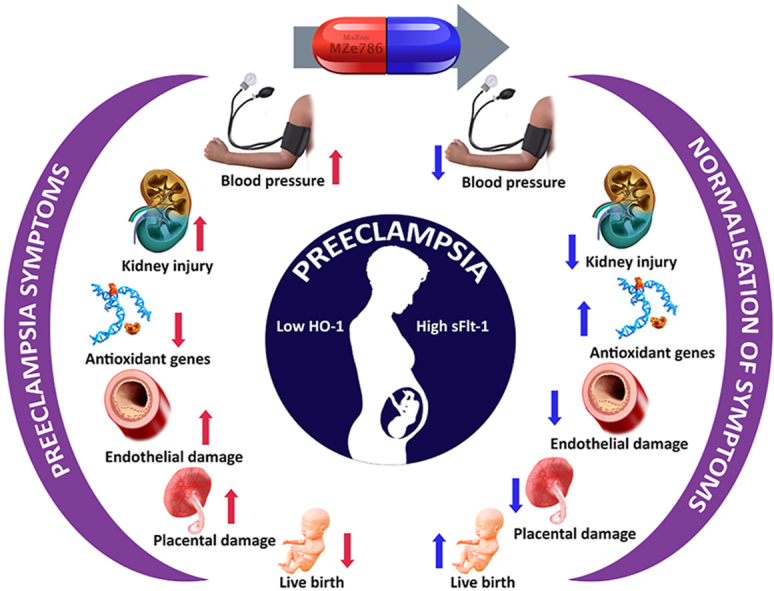

## 1 Introduction

Preeclampsia is a systemic disorder of pregnancy, affecting over 10 million women and annually accounts for over 76,000 maternal deaths and 500,000 infant deaths [[1], [2], [3]]. This equates to a life lost every minute. Clinically, preeclampsia manifests itself as de novo onset of hypertension during pregnancy along with one or more of the complications affecting heart, lungs, kidney, liver and feto-placental systems. Importantly, women who have experienced preeclampsia are three to four times more at risk of developing high blood pressure later in life; twice as likely to develop heart disease, heart failure, stroke [[4], [5], [6]] and they are three times more likely to develop vascular dementia later in life [7]. There are no effective pharmacological agents to prevent or treat preeclampsia. In women at high risk of developing preeclampsia, aspirin appears to reduce the risk of preeclampsia by 15% if taken from 12 weeks of pregnancy [8]. The only solution to protect the life of the mother is the delivery of the baby with the placenta. However, induced preterm birth jeopardises the life and health of the infant both in the short-term and long-term [9].

Several studies have validated the hypothesis that preeclampsia arises due to ‘increase in the level of endogenous soluble Flt-1 (sFlt-1) that may antagonize the beneficial effects of vascular endothelial growth factor (VEGF)’[[10], [11], [12], [13]]. Rodents treated with adenovirus encoding sFlt-1 develop preeclampsia-like phenotype with high blood pressure and kidney damage [12,14]. The most prominent factor linked to the pathogenesis of preeclampsia is the elevated level of sFlt-1 [15]. Although high maternal sFlt-1 is unique to preeclampsia, not all women with high sFlt-1 develop preeclampsia [16].

Human heme oxygenase-1 (Hmox1/HO-1) deficiency leads to severe and persistent endothelial damage [17], which is a hallmark of preeclampsia. The importance of HO-1 in preeclampsia was first highlighted when induction of HO-1 was shown to attenuate tumour necrosis factor alpha-induced placental damage *ex vivo* [18]. Adenoviral overexpression of HO-1 inhibits sFlt-1 expression in endothelial cells [19] and HO-1 is decreased in the placenta from preeclamptic women compared to healthy pregnancy [18]. More recent studies have confirmed the significance of this protective enzyme [20]. First trimester chorionic villous (fetal placental cells) sampling revealed a reduction in Hmox1 mRNA from women who subsequently went on to develop preeclampsia compared to normal pregnancy [21]. Furthermore, a recent study showed that guanine-thymine (GTn) microsatellite in the Hmox1 promoter decrease HO-1 expression and there was an association between long fetal and maternal GTn repeats and lower placental and serum HO-1 levels, indicating that partial loss of HO-1 activity may increase the risk of preeclampsia [22]. Indeed, HO-1 expression increases by 15-folds during normal pregnancy and plays a crucial role in the healthy development of pregnancy [[22], [23], [24]].

In women with preeclampsia, the expression of hydrogen sulfide producing enzyme, cystathionine gamma-lyase (CSE) is decreased in the placenta and so is the levels of hydrogen sulfide (H_2_S) in the maternal circulation [25]. Pregnant C57BI6/J mice treated with a CSE inhibitor, dl-Propargylglycine (PAG), develop ‘preeclampsia phenotype’, characterised by hypertension, kidney damage and fetal growth restriction (FGR) [25]. H_2_S is a potent promoter of vasodilatation [26], exhibits cytoprotective anti-inflammatory properties [27], protects against reperfusion injury [28] and stimulates angiogenesis [29]. Therapeutic potential of H_2_S for its anti-inflammatory and cytoprotective properties have been explored through preservation of mitochondrial function and regulation of calcium homeostasis [30,31].

The current study demonstrates that the partial loss of HO-1 in a high sFlt-1 environment leads to preeclampsia. Overexpression of sFlt-1 in HO-1 haploid deficient (Hmox1^+/−^) pregnant mice showed exacerbated increase in blood pressure, kidney damage, altered placental morphology and adverse fetal outcomes. The H_2_S-releasing aspirin, MZe786 suppressed these adverse events. Finally, we demonstrated that the MZe786 is a better candidate at preventing preeclampsia than aspirin or H_2_S alone.

## 2 Materials and method

For detailed descriptions of the materials and methods used, please see Supplementary file.

## 3 Materials

The H_2_S-releasing molecules, MZe786 (2-acetyloxybenzoic acid 4-(3-thioxo-3*H*-1,2-dithiol-5-yl)phenyl ester) and MZe486 (5-(4-hydroxyphenyl)-1,2-dithiol-3-thione) are shown to be safe and effective with a defined pharmacological profile [32].

## 4 Methods

### 4.1 Animal experimental protocol

Pregnant mice were injected with adenovirus encoding sFlt-1 (Ad-sFlt-1) or cytomegalovirus control virus (Ad-CMV) (Vector Biolabs, USA) at 1x10^9^ plaque forming unit (PFU) via tail vein injection on day E11.5. The Hmox1^+/−^ mice were separated into different treatment groups and treated with: (A) Drug Carrier (Control), (B) 50 mg/kg MZe786, (C) 29/mg/kg ADTOH and (D) 23 mg/kg aspirin up to E17.5. The mice were placed in metabolic cages and urine was collected for 24 h from E16.5 to E17.5, at which time, tissues were harvested.

### 4.2 Blood pressure and ultrasound measurements

Blood pressure was measured on E17.5 as described previously [25]. In addition, ultrasound analysis of uterine and umbilical arteries was conducted on E17.5 (see Supplementary file for details).

### 4.3 Enzyme linked immunosorbent assay

Enzyme-linked immunosorbent assay (ELISA) kits for murine sVEGFR1/Flt-1, KIM-1, sEng and E-selectin were obtained from R&D Systems and performed according to the manufacturer's specifications.

### 4.4 Quantitative polymerase chain reaction

RNA was extracted from the kidneys using the RNeasy minikit (Qiagen, Germany) and real-time PCR was performed as previously described [33].

### 4.5 Tissue processing and histological analysis

Murine renal and placental tissues were stained with H&E and were imaged using NanoZoomer (Hamamasu, Japan). The area of the labyrinth zone was measured and analysed using ImageJ. The damaged glomeruli were counted and calculated.

### 4.6 Trimethylsulfonium measurement

Trimethylsulfonium was measured in the urine using HPLC method (see Supplementary file).

### 4.7 Statistical analysis

Data is presented as either representatives, mean and SEM or median and range as appropriate. Comparison between two groups was performed using Mann-Whitney *U* test (non-parametric). Comparisons among three or more groups were performed using One-Way or Two-Way ANOVA. Statistical analysis was performed using GraphPad Prism 8.1 software (GraphPad Software, La Jolla, CA). Statistical significance was set at p < 0.05.

## 5 Results

### 5.1 Hmox1 deficient mice show severe preeclampsia symptoms in the presence of high sFlt-1

**Fig. 1:**
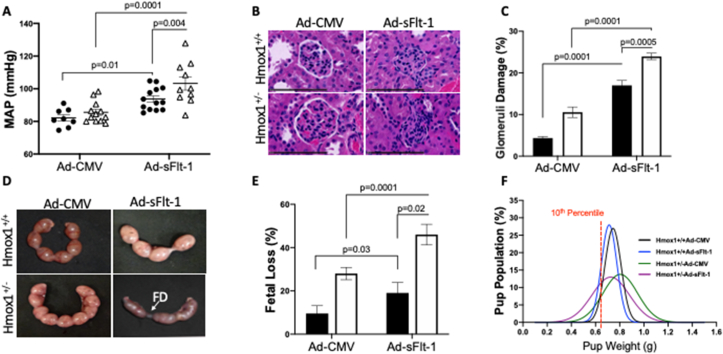
**High sFlt-1 causes a severe preeclampsia phenotype in Hmox1 haploid deficient mice. (A)** Mean arterial blood pressure (MAP) measured at E17.5 gestation in Hmox1^+/+^ (Black circles) and Hmox1^+/−^ (black triangle) mice after administration of Ad-CMV (control) or Ad-sFlt-1 (1x10^9^ pfu) at E11.5. **(B)** Serial sections of representative glomeruli stained with hematoxylin and eosin (HE). Scale bars, 100 μm. **(C)** Single-blind analysis of glomeruli damage from Hmox1^+/+^ (Black column) and Hmox1^+/−^ (White column) mice treated with Ad-CMV or Ad-sFlt-1. (n = 6 Hmox1^+/+^; n = 6 Hmox1^+/−^). **(D)** Representative images of uterine horn (E17.5) showing overexpression of sFlt-1 leads to fetal death (FD). **(E)** Fetal loss expressed as a percentage of the total pup numbers. **(F)** Fetal weight distribution of pups and 10th percentile pup weight population mark, representing the fetal growth restriction in the weight curve from Hmox1^+/+^ and Hmox1^+/−^ mice treated with Ad-CMV or Ad-sFlt-1. (n = 13 Hmox1^+/+^; n = 14 Hmox1^+/−^). Results are expressed as representative or as mean (**±**SEM). Analysed by Two-way ANOVA.

Maternal hypertension and kidney damage are the key clinical features of preeclampsia. To determine whether HO-1 haploid deficient mice (Hmox1^+/−^) in the presence of high sFlt-1 cause preeclampsia-like symptoms, Hmox1^+/+^ and Hmox1^+/−^ pregnant mice were injected with either Ad-CMV or Ad-sFlt-1 via tail vein injection on day E11.5. Ad-sFlt-1 injection resulted in a significantly higher mean arterial pressure (MAP) in Hmox1^+/+^ mice and in Hmox1^+/−^ mice compared to the CMV treated counterparts. More importantly, MAP was significantly higher in Hmox1^+/−^ compared to Hmox1^+/+^ mice following Ad-sFlt-1 injection (Fig. 1A).

Glomerular endotheliosis results in poor filtration and increased protein in the urine is seen in preeclampsia [34]. HO-1 deficiency and high circulating level of sFlt-1 caused glomerular degeneration and narrowing or disappearance of Bowman's space (Fig. 1B). This was confirmed by randomized single-blind scoring of kidney sections, which showed significantly higher levels of glomeruli damage in mice treated with Ad-sFlt-1 both in Hmox1^+/+^ and Hmox1^+/−^. The glomeruli damage was further exacerbated by Ad-sFlt-1 in Hmox1^+/−^ mice compared to Hmox1^+/+^ mice (Fig. 1C).

Poor fetal outcomes and FGR are linked to preeclampsia [35]. FGR is where the fetus is smaller than expected at gestational age and is often described as an estimated weight less than the 10th percentile [36]. To investigate the impact of high circulating level of sFlt-1 on fetus, fetal loss and fetal weight were assessed. Fig. 1D shows representative images of unhealthy-looking uterine horn and signs of fetal death (FD) by fetal reabsorption in Hmox1^+/−^ pregnant mice injected with Ad-sFlt-1. Fetal loss expressed as a percentage increased in Hmox1^+/+^ Ad-sFlt-1 injected mice (17.8%) compared to Hmox1^+/+^ mice injected with Ad-CMV (9.5%). Fetal loss was further increased in Hmox1^+/−^ mice injected with Ad-sFlt-1 (46.0%) compared to Hmox1^+/−^ mice injected with Ad-CMV (27.9%) (Fig. 1E). In addition to fetal death, fetal weight was related to the genotype and the levels of sFlt-1. In the 10th percentile population, pups from Hmox1^+/+^ mice injected with Ad-CMV were 10.71% of the total population, which increased to 16.85% with Ad-sFlt-1 injections (Supplementary Table S1). Hmox1^+/+^ mice also had a steeper distribution compared to Hmox1^+/−^, which was much shallower; Ad-sFlt-1 injection shifted both curves to the left (Fig. 1F). A larger percentage of the Hmox1^+/−^ mice pups were growth restricted compared to Hmox1^+/+^ mice (33.33% of Hmox1^+/−^ mice injected with Ad-sFlt-1 were growth restricted compared to 13.51% when injected with Ad-CMV, Supplementary Table S1). The data shows that Hmox1^+/−^ pregnant mice under a high sFlt-1 environment serve as a useful model to evaluate therapeutics that may suppress the clinical features of preeclampsia.

### 5.2 Hydrogen sulfide releasing molecule rescued maternal outcome in Hmox1^+/−^ pregnant mice in a high sFlt-1 environment

**Fig. 2:**
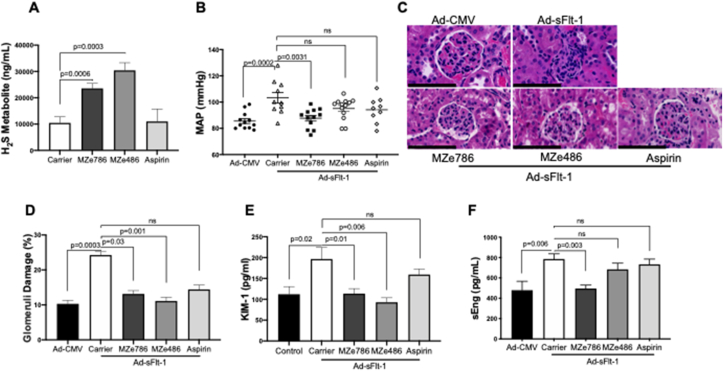
**MZe786 rescues maternal outcome in Hmox1**^**+/−**^**pregnant mice in a high sFlt-1 environment. (A)** H_2_S metabolite, trimethyl sulfonium (TMS) levels measured in urine on day E17.5 in Hmox1^+/−^ timed pregnant mice injected Ad-sFlt-1 and treated with Carrier (control), MZe786, MZe486 or aspirin. **(B)** Mean arterial blood pressure (MAP) recorded at E17.5 gestation in Hmox1^+/−^ timed pregnant mice injected with Ad-CMV or Ad-sFlt-1 and treated with MZe786, MZe486 or aspirin. **(C)** Representative serial sections of glomeruli stained with hematoxylin and eosin (H&E). Scale bars, 100 μm. **(D)** Single-blind analysis of glomeruli damage. **(E)** Urinary kidney injury marker −1 (KIM-1) level. **(F)** Plasma sEng level measured on day E17.5 gestation in Hmox1^+/−^ timed pregnant mice injected with Ad-CMV or Ad-sFlt-1 and treated with carrier, MZe786, MZe486 or aspirin (n = 11 Ad-CMV; n = 13 Carrier; n = 13 MZe786; n = 14 MZe486; n = 10 Aspirin group). Results are expressed as representative or as mean (**±**SEM). Analysed by One-way ANOVA.

To evaluate the therapeutic potential of H_2_S-releasing aspirin, MZe786, in preeclampsia, Hmox1^+/−^ mice were injected with Ad-sFlt-1 on day E11.5 and simultaneously given four treatment regimens (drug carrier, MZe786, MZe486 or aspirin). Firstly, to prove that MZe786 releases H_2_S, the methylated metabolite of H_2_S, trimethylsulfonium (TMS) was measured in the urine of Hmox1^+/−^ mice by mass spectroscopy. TMS was significantly increased in the MZe786 and MZe486 treated animals indicating an increase in the H_2_S pool by the H_2_S-releasing drugs (Fig. 2A). MZe786 significantly reduced MAP in Hmox1^+/−^ pregnant mice exposed to high levels of sFlt-1 (Fig. 2B). Interestingly, no significant changes were seen in MZe486 and aspirin-treated Hmox1^+/−^ mice (Fig. 2B). Representative H&E staining showed severe glomerular damage in Hmox1^+/−^ pregnant mice treated with Ad-sFlt-1 compared to Ad-CMV treatment (Fig. 2C). Administration of H_2_S-releasing molecules rescued glomerular damage. This was quantified using randomized single-blind scoring of kidney sections which showed a significant reduction in the levels of glomeruli damage in mice administered with MZe786 and MZe486 under high sFlt-1 conditions (Fig. 2D). Kidney Injury Molecule-1 (KIM-1) is a marker of acute kidney injury [37]. Overexpression of sFlt-1 significantly increased urinary KIM-1 level in Hmox1^+/−^ pregnant mice compared to Ad-CMV treated animals and administration of H_2_S-releasing molecules returned KIM-1 to normal levels (Fig. 2E). Surprisingly, mice treated with aspirin showed only a slight but not significant reduction in glomerular damage and KIM-1 levels in the presence of high sFlt-1 (Fig. 2C–E).

Soluble endoglin (sEng) is a marker of endothelial activation and is increased in preeclampsia [38,39]. Ad-sFlt-1 injection significantly increased circulatory levels of sEng in Hmox1^+/−^ pregnant mice compared to Ad-CMV injected counterparts and concomitant administration of MZe786 significantly reduced sEng. However, MZe486 and aspirin did not reduce sEng in these mice (Fig. 2F). The levels of E-selectin, a circulating adhesion molecule were also tested to evaluate endothelial activation in Hmox1^+/−^ pregnant mice under a high sFlt-1 environment. Hmox1^+/−^ mice treated with Ad-sFlt-1 increased plasma E-selectin while administration of MZe786, MZe486 and Aspirin reduced E-selectin (Supplementary Fig. S1A). The drugs administered had no interference with sFlt-1 production in these mice as the circulating level of sFlt-1 measured on day E17.5 consistently showed a significant increase in Ad-sFlt-1 injected mice (Supplementary Fig. S1B) and there was no significant change in the circulating levels of VEGF in any of the treatment groups (Supplementary Fig. S1C).

### 5.3 Hydrogen sulfide releasing molecule rescued fetal outcome in Hmox1^+/−^ pregnant mice in a high sFlt-1 environment

**Fig. 3:**
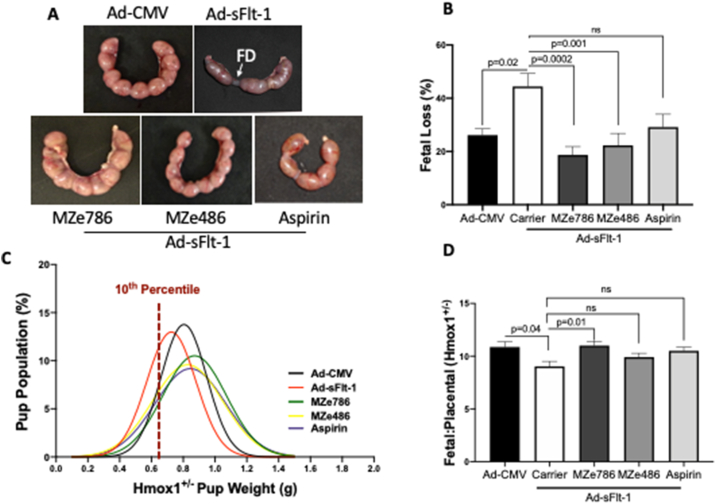
**MZe786 improves fetal outcome in a high sFlt-1 environment in Hmox1**^**+/−**^**mice. (A)** Representative images of uterine horn (E17.5) showing rescue of fetal death (FD) in sFlt-1 induced Hmox1^+/−^ mice by H_2_S-releasing molecules. **(B)** Fetal loss expressed as a percentage over total pup numbers in Hmox1^+/−^ mice injected with Ad-CMV or Ad-sFlt-1 and treated with MZe786, MZe486 or aspirin. **(C)** Hmox1^+/−^ fetal weight distribution and 10th percentile pup weight population mark, representing the fetal growth restriction in the weight curve **(D)** Hmox1^+/−^ Fetal:placental ratio in Hmox1^+/−^ mice injected with Ad-CMV or Ad-sFlt-1 and treated with MZe786, MZe486 or aspirin. Results are expressed as representative or as mean (**±**SEM). Analysed by One-way ANOVA.

Fetuses with FGR are at risk of poor long-term health outcomes, such as cardiovascular and endocrine disease in adulthood [40] and neurological development [41]. To evaluate the effect of H_2_S-releasing aspirin, MZe786, on the fetal outcome, fetal loss and fetal weights were assessed on E17.5. Fig. 3A shows representative images of healthy-looking uterine horns in Hmox1^+/−^ pregnant mice when treated with H_2_S-releasing molecules under high levels of sFlt-1. Overexpression of sFlt-1 led to 44.4% fetal loss in Hmox1^+/−^ mice compared to 26.1% in Ad-CMV injected mice. Administration of MZe786 and MZe486 significantly reduced the fetal loss to 18.6% and 22.2%, respectively (Fig. 3B). A slight but not significant reduction in the fetal loss was also observed in aspirin-treated mice (Fig. 3B). Administration of MZe786, MZe486 and aspirin decreased FGR from 33.3% to 14.23%, 19.2% and 22.2%, respectively (Fig. 3C, Supplementary Table S2).

Abnormal development of the placenta has long been associated with abnormal fetal outcome [13]. The labyrinth zone is made of two layers of multinucleated syncytiotrophoblast cells where the exchange of nutrients and waste takes place between the mother and the fetus [42]. Double-blinded histological analysis of the placental sections showed that overexpression of sFlt-1 significantly reduced labyrinth zone area in Hmox1^+/−^ pregnant mice compared to Ad-CMV injected mice. Oral administration of MZe786 increased labyrinth zone area. No changes were observed in MZe486 or aspirin treated mice (Supplementary Fig. S2). Interestingly, there were no changes to the resistance index of the uterine or the umbilical artery measurements using ultrasound following these different treatment regiments (Supplementary Fig. S3). The Fetal:Placental ratio is a health indicator reflecting the balance between fetal and placental growth [43]. The ratio was significantly reduced in the high sFlt-1 environment and this was rescued by administration of MZe786 in Hmox1^+/−^ mice. No changes in Fetal:Placental ratio were seen in MZe486 or aspirin-treated Hmox1^+/−^ pregnant mice in high sFlt-1 conditions (Fig. 3D).

### 5.4 MZe786 stimulates antioxidant genes in a high sFlt-1 environment in Hmox1^+/−^ pregnant mice

**Fig. 4:**
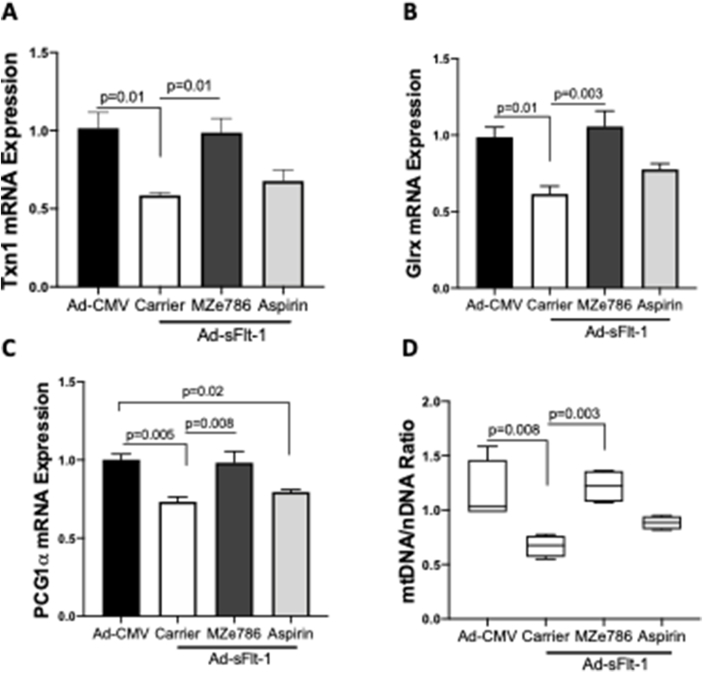
**MZe786 stimulates antioxidant defence in Hmox1**^**+/−**^**mice overexpressing sFlt-1. (A)** Kidney relative mRNA expression of genes; **(B)** Txn1, **(C)** Glrx and **(D)** PGC1α measured by quantitative PCR n Hmox1^+/−^ mice injected with Ad-CMV or Ad-sFlt-1 and treated with carrier, MZe786 or aspirin. **(E)** Mitochondrial content measured as the ratio of mtDNA/nDNA in Hmox1^+/−^ mice injected with Ad-CMV or Ad-sFlt-1 and treated with carrier, MZe786 or aspirin. Results are expressed as representative or as mean (**±**SEM). Analysed by One-way ANOVA.

Soluble Flt-1 is reported to exacerbate mitochondrial reactive oxygen species formation and mitochondrial membrane potential dissipation in endothelial cell [44,45]. As high sFlt-1 induced kidney injury, we sought to investigate if low HO-1 and high sFlt-1 compromise the expression of antioxidant genes and whether MZe786 or aspirin could rescue this damage. Adenoviral overexpression of sFlt-1 in Hmox1^+/−^ pregnant mice significantly reduced the transcription of antioxidant defence genes in the kidney; thioredoxin (Txn1) (Fig. 4A) and glutaredoxin-1 (Glrx) (Fig. 4B). Oral administration of MZe786 significantly restored the transcription of Txn1 and Glrx (Fig. 4A and B**)**. Peroxisome proliferator-activated receptor gamma coactivator 1-alpha (PGC1-α), a master regulator of metabolism and antioxidant defence system, was also reduced in Hmox1^+/−^ pregnant mice injected with Ad-sFlt-1 which was restored by the administration of MZe786 (Fig. 4C). Accordingly, MZe786 restored the content of mitochondria as measured by the ratio of mitochondrial DNA and nuclear DNA (mtDNA/nDNA) (Fig. 4D). Aspirin alone did not restore the transcription of Txn1 and Glrx and did not improve the mitochondrial biogenesis signal or mitochondrial content in the kidney of Hmox1^+/−^ pregnant mice injected with Ad-sFlt-1 (Fig. 4). These results suggest that MZe786 may regulate the content of mitochondria by modulating the transcription and expression of PGC1α.

## 6 Discussion

Earlier studies have demonstrated that HO-1 is a negative regulator of sFlt-1 [19] and is decreased in preeclampsia [18]. Overexpression of sFlt-1 leads to high blood pressure, kidney damage and fetal growth restriction as previously reported in pregnant rat [46] and mice [47]. In a low HO-1 environment, the adverse outcomes associated with overexpression of sFlt-1 were further aggravated with an increase in blood pressure, kidney damage and fetal death. Partial loss of HO-1 alone did not show visible features of preeclampsia in these pregnant mice; however, these clinical phenotypes were only present and exacerbated when sFlt-1 levels were high. Our data demonstrate that preeclampsia arises due to a double hit, a combination of decreased HO-1 activity and increased sFlt-1 levels leading to systemic endothelial activation and organ damage.

The National Institute of Health and Care Excellence's (NICE) recommends that women at high risk of developing preeclampsia should take 75–150 mg of aspirin daily from 12 weeks of pregnancy until delivery [48]. Although aspirin only reduces the risk of preeclampsia by 15%, it does so without changing the incidence of FGR [8,49]. Furthermore, there is no therapeutic available to prevent preeclampsia. The only way to prevent a life-threatening seizure developing due to eclampsia, is the premature delivery of the baby with the placenta.

The lack of an effective therapeutic to prevent or treat preeclampsia is responsible for an annual global cost burden of GBP 76.6 billion [50]. In the preeclamptic model where sFlt-1 levels are high and HO-1 activity is low, we show that aspirin is unable to prevent preeclampsia by inhibiting hypertension or kidney injury or preventing fetal loss. It is known for over a decade that H_2_S and its precursors are potent inducers of HO-1 [51,52]. A novel approach to prevent preeclampsia was to develop a chemically modified aspirin. MZe786, 2-acetyloxybenzoic acid 4-(3-thioxo-3H-1,2-dithiol5-yl)phenyl ester is a novel molecule (also known as ACS14) comprising of an H_2_S-releasing dithiole-thione moiety (MZe486, also named ADTOH) attached by an ester linkage to aspirin [32]. It protects the gastric mucosa via its anti-cyclooxygenase activity. It is able to suppress thromboxane synthesis, to decrease 8-isoprostane levels and homocysteine levels as well as to increase plasma and tissue glutathione (GSH) levels. In addition, MZe786 produces a concentration-dependent increase in Hmox1 promoter activity in NIH3T3-HO-1-luc cells whereas aspirin had no significant increase on Hmox1 promoter activity in these cells [32]. It also exerted cardiovascular protection in a buthionine sulfoximine (GSH synthase inhibitor) model of metabolic syndrome [53]. These effects of MZe786 may help to balance the redox system.

H_2_S-releasing aspirin, MZe786, successfully reduced blood pressure, kidney damage and improved fetal outcome in Hmox1^+/−^ mice under high levels of sFlt-1 in part, by increasing the release of H_2_S as its' metabolite, TMS, is increased in MZe786 treated group and not in the aspirin group. The presence of TMS in the urine is a good indicator of the presence of H_2_S in the kidney and circulation. The potential of MZe786 and MZe486 to release H_2_S in circulation has been previously reported by Giustarini et al., 2010. Administration of MZe786 and MZe486 increased Cysteine and GSH levels in many organs, including the kidney [54]. This is a good indicator of H_2_S release and MZe786's ability to modulate thiol homeostasis to offer protection and reduce oxidative stress and may explain the inhibitory effect of MZe786 on KIM-1 and sEng suppression.

Our results provide novel insights into the role of HO-1 in modulating the antioxidant gene expression and suggest MZe786 protects the renal antioxidant capacity. Our study demonstrates that MZe786 improves the renal antioxidant capacity via upregulation of Trx-1 and Glrx genes transcription. Several mitochondrial dysregulation pathways have been associated with endothelial dysfunction including, PGC-1α, a transcriptional coactivator linked to mitochondrial biogenesis and antioxidant defense. Overexpression of sFlt-1 reduced PGC-1α gene transcription and mitochondrial content in Hmox1^+/−^ pregnant mice in comparison to Ad-CMV injected mice. This is in line with our recent publication, which demonstrated that overexpression of sFlt-1 reduced transcription of cardiac antioxidant genes in non-pregnant Hmox1^+/−^ mice. In addition, sFlt-1 led to inhibition of cardiac mitochondrial activity and administration of MZe786 increased antioxidant genes and rescued mitochondrial activity by stimulating cardiac mitochondrial biogenesis [33]. These studies illustrate that despite the presence of high sFlt-1, MZe786 nullified the effect of high sFlt-1 and low HO-1 due to its ability to increase the expression of antioxidant genes.

H_2_S promotes angiogenesis by upregulation of nitric oxide (NO) [55] and H_2_S donors restore placental angiogenesis in pregnant mice under reduced CSE activity [25]. VEGF is known to stimulate NO release [56]. Interestingly, there was no significant change in the circulating levels of VEGF in any of the treatment groups. MZe786 may have an impact on the VEGF signalling at a cellular level. H_2_S is known to stimulate tissue VEGF expression, which activates endothelial nitric oxide synthase (eNOS) and suppresses oxidative stress [57]. MZe786 ameliorate placental structural alteration as indicated by increased labyrinth zone in Hmox1^+/−^ mice treated with Ad-sFlt-1. Reduced labyrinth zone is associated with FGR; a small labyrinth has reduced transport surface area and thereby limiting the nutrient available to the fetus [58,59]. In agreement, we see the highest percentage of growth-restricted pups in Hmox1^+/−^ mice treated with Ad-sFlt-1 and administration of MZe786 reduces the population of growth-restricted pups back to normal. Importantly, H_2_S-releasing molecules, MZe786 and MZe486 decreased fetal death induced by overexpression of sFlt-1 in Hmox1^+/−^ mice. Interestingly. there were no changes in the resistance index of the uterine or the umbilical artery measured using ultrasound following treatment indicating that alteration in uterine artery hemodynamic are not required for preventing preeclampsia or improving fetal outcome.

Earlier study showed that inhibition of CSE activity in human placental explants from the first trimester (8–12 weeks) of pregnancy caused a decrease in placental growth factor (PlGF) production [25]. The ratio sFlt-1/PlGF is referred to in human pregnancy as a potential indicator of preeclampsia. Unfortunately, this is not the same for mouse models as the mouse only expresses PlGF-2 transcript [60] and the protein product is heparin-bound [61]. This means that free PlGF in the murine circulation cannot be detected by ELISA to reflect a similar scenario as observed in humans. Furthermore, preeclampsia is defined by clinical characteristics. The sFlt-1/PlGF ratio has false positives and false negatives, it is used as a rule-out test and not as a rule-in test [62]. The improvement in the clinical parameters with the MZe786 clearly demonstrates that the pathology of preeclampsia is halted due to the given compound.

In conclusion, overexpression of sFlt-1 in HO-1 compromised pregnant mice exhibit the clinical features of preeclampsia with a significant increase in blood pressure, kidney damage and fetal growth restriction and fetal loss. All these symptoms were prevented by the administration of the H_2_S-releasing aspirin, MZe786 and this offers a novel therapeutic strategy over aspirin and is worthy of clinical exploration.

## Declaration of competing interest

HR, SA, LSA, IHKD, FAA, SwA and AS declare they have no conflict of interest. AA is the Executive Chairman and the majority shareholder in MirZyme Therapeutics. AA and KW are inventors for the use of hydrogen sulphide compounds in the treatment of preeclampsia (WO2014132083A2).
