# Supplementary material for: MZe786, a hydrogen sulfide-releasing aspirin prevents preeclampsia in heme oxygenase-1 haplodeficient pregnancy under high soluble flt-1 environment
Source: Redox Biol. 2020 Oct 24;38:101768. doi: 10.1016/j.redox.2020.101768 (PMC7610044; doi:10.1016/j.redox.2020.101768)
Supplement: Multimedia component 1 [file mmc1.pdf]

MZe786, a hydrogen sulfide-releasing aspirin prevents preeclampsia in heme oxygenase-1 haplodeficient pregnancy under high soluble flt-1 environment

Homira Rezai[1,2*], Shakil Ahmad[2*], Faisal A. Alzahrani[1,3], Lissette Sanchez-Aranguren[1,2], Irundika HK Dias[2], Swati Agrawal[1,4], Anna Sparatore[5], Keqing Wang[2], Asif Ahmed[1,2,3,6]

[1]Mirzyme Therapeutics, Innovation Birmingham Campus, Faraday Wharf, Holt Street, Birmingham B7 4BB, United Kingdom. [2]Aston Medical Research Institute, Aston Medical School, Birmingham, United Kingdom. [3]Department of Biochemistry, ESC Research Unit, Faculty of Science, King Fahd Medical Research Center, King Abdulaziz University, Jeddah 21589, Saudi Arabia. [4]Mt Sinai Hospital, University of Toronto, Toronto, Canada. [5]Department of Pharmaceutical Sciences, University of Milan, Milan, Italy. [6]President's Office, University of Southampton, Southampton, SO17 1BJ, UK.

Short title: MZe786 rescue preeclampsia in mice

Keywords: Preeclampsia, Hydrogen Sulfide, heme oxygenase-1, sFlt-1, hypertension

For Correspondence:

Prof Asif Ahmed (asif.ahmed@mirzyme.com and @ProfAsifAhmed)

Mirzyme Therapeutics Limited,

Innovation Birmingham Campus, Faraday Wharf, Holt Street,

Birmingham B7 4BB, U.K.

Materials and Method

Animal Experimentation

All animal experiments were carried out using procedures approved by the Aston University Ethical Review Committee in compliance with the UK Home Office Licence Number 3003453 in accordance with the 'Guidance on the operation of Animals' under the United Kingdom Animals (Scientific Procedures) Act 1986.

Materials

The H_2S-releasing molecules, MZe786 (2-acetyloxybenzoic acid 4-(3-thioxo-3*H*-1,2-dithiol-5-yl)phenyl ester) and MZe486 (5-(4-hydroxyphenyl)-1,2-dithiol-3-thione) are shown to be safe and effective with a defined pharmacological profile (1). Aspirin was purchased from Sigma, UK. Drugs were prepared in 0.05% carboxymethylcellulose (drug carrier).

Adenovirus preparation

Recombinant adenovirus encoding mouse sFlt-1 under the control of the CMV promoter (Ad-sFlt-1) and Adenovirus containing the CMV promoter (Ad-CMV), was purchased from Vector Biolab (U.S) and stored at -80°C.

Animal experimental protocol

Heterozygrous female Hmox1$^{+/-}$ mice between the ages of twelve and twenty-five-weeks were time mated with similar age male Hmox1$^{+/-}$ mice. Female Hmox1$^{+/+}$ mice between the ages of twelve to twenty-five-weeks were time mated with similar age male Hmox1$^{+/+}$ mice. The first day of pregnancy (E0.5) was defined by the presence of a vaginal plug. Pregnant mice were injected with adenovirus encoding sFlt-1 (Ad-

sFlt-1) or control virus (Ad-CMV) (Vector Biolabs, USA) at 1x 10^9 plaque forming unit (PFU) via tail vein injection on day E11.5. The Hmox1$^{+/-}$ mice were then separated into different treatment groups and were treated with the following: (A) Drug Carrier (Control), (B) 50mg/kg ACS14, (C) 29/mg/kg ADTOH and (D) 23mg/kg Aspirin from day E11.5 to E17.5. On E16.5, the mice were placed in metabolic cages to collect urine for 24 hours. On day E17.5 the mice were sacrificed, and the tissues were harvested.

Blood pressure measurement and tissue collection

Blood pressure was measured on E17.5 as previously described (2). Following blood pressure measurements, blood samples were collected via cardiac puncture using vacutainer tubes containing EDTA. The animals were euthanized, and their kidneys, livers, and placentas were collected and fixed in 4% paraformaldehyde for histology. Using fine dissection forceps, the individual yolk sac containing the embryos was separated from the uterus. The yolk sacs were further dissected to separate the placenta and embryo. The total number of live and resorbed fetuses and placentas were counted, weighed and dissected using fine forceps under a stereomicroscope.

Ultrasound of uterine and umbilical arteries

On day E17.5, mice were anesthetised with 2% isoflurane and placed in a supine position on a heated mat for ultrasound imaging and was gently stabilised by each paw being taped to ECG electrode covered with conductance gel. The heart rate was maintained at 450 ± 50b/min. Ophthalmic ointment to the eye was applied to prevent corneal drying. A rectal probe was inserted to monitor core body temperature which was maintained in the rage of 36±1°C. Abdomen hair was removed using hair removal cream (Veet Cream, Boots, UK) and was cleaned with wetted gauze to make sure no trace of the shaving gel remained on

mice. Pre-warmed ultrasound gel was applied on the abdomen avoiding any bubbles to be induced into the scanning area. Images were obtained using Vevo® 3100 ultrasound system (Visual Sonics, Amsterdam, Netherlands). Ultrasound probe with 40 MHz frequency was fixed and mobilised with a mechanical holder. For uterine artery imaging, the bladder was first identified to use as a reference point to locate the uterine artery. Once the uterine artery flow was located using colour Dopplar mode, and the velocities of the uterine arteries were recorded. For umbilical artery imaging, using B Mode, umbilical arteries were visualised in an intra-amniotic segment in the longitudinal plane of the fetoplacental unit. Colour Doppler and PW Doppler modes were applied, and the velocities were recorded. The resistance index (RI) was calculated using Vevo® Labs software.

Enzyme linked immunosorbent assay

Enzyme-linked immunosorbent assay (ELISA) kits for murine sVEGFR1/Flt-1, KIM-1, sEng and E-selectin were obtained from R&D Systems and performed according to the manufacturer’s specifications.

Quantitative polymerase chain reaction

RNA was extracted from the kidney using the RNeasy minikit (Qiagen, Germany) and real-time PCR was performed as previously described (3).

Tissue processing and histological analysis

Murine kidney and placental tissues were fixed in 4% paraformaldehyde and were processed, embedded with paraffin and stained with H&E by contract research laboratory, Histologix, UK. H&E stained kidney and placenta tissues were imaged using NanoZoomer (Hamamasu, Japan). The area of the labyrinth zone was measured and analysed using ImageJ. Briefly, a virtual grid of fixed-sized

intersecting lines was overlaid on the kidney images using NDP.View. Each glomerulus in these grids was counted and blindly assessed. The damaged glomeruli were then calculated.

Trimethylsulfonium measurement

TMS is a well-recognized metabolite of H2S, the reason we chose TMS was precisely due to the plethora of prior publications that support TMS as a specific indicator of steady state levels of tissue H2S (4-6). Urine collected from pregnant Hmox1$^{+/-}$ mice treated with placebo, MZe786, MZe486 and aspirin was vortexed for 30 seconds prior to filtration through 0.2µm nylon filters (Fisher Scientific, UK). The samples were diluted 1:10 in samples buffer containing 80% acetonitrile with 10mM ammonium formate. Commercially available trimethylsulfonium (TMS) iodide and isotopically labelled d9-TMS, were analysed using liquid chromatography (LC, DIONEX UltiMate 3000, Thermo Scientific UK Ltd., Hemel Hempstead) on-line coupled to the ESI-QqLIT-MS/MS. TMS and d9-TMS were individually injected to identify optimal fragments based on their abundance for multiple reactions monitoring (MRM) analysis. The precursor ion scans were performed between 40 m/z to 100 m/z mass range with ESI-MS in a positive ion mode. At least three diagnostic product ions were selected for each analyte, and collision energy, depolarisation potential and exit potential was optimised for each transition pair. 10µl urine samples in sample buffer were separated on a cation exchange column using mobile phase, at 25°C. The flow rate was maintained at 250µl/min. Acquired data were processed using Analyst Software (version 1.6.2, AB Sciex).

Statistical analysis

Data is presented as either representatives, mean and SEM or median and range as appropriate. Comparison between two groups was performed using Mann-Whitney U-test (non-parametric). Comparisons among three or more groups were performed using One-Way or Two-Way ANOVA. Statistical analysis was performed using GraphPad Prism 8.1 software (GraphPad Software, La Jolla, CA). Statistical significance was set at $p<0.05$.

Supplementary Table S1. The 10th percentile pup population.

Genotype	Injection	10th Percentile population (%)
Hmox1$^{+/+}$	Ad-CMV	10.7
	Ad-sFlt-1	16.8
Hmox1$^{+/-}$	Ad-CMV	13.5
	Ad-sFlt-1	33.3

Supplementary Table S2. The 10th percentile pup population following different treatment regimens.

Injection	Treatment	10th Percentile population (%)
Ad-CMV	Carrier	16.1
Ad-sFlt-1	Carrier	33.3
	MZe786	14.3
	MZe486	19.2
	Aspirin	22.2

Supplementary Figure Legends

Supplementary Figure S1. (A) E-selectin levels and **(B)** Plasma sFlt-1 levels and **(C)** plasma VEGF-A levels at day E17.5 in Hmox1$^{+/-}$ pregnant mice. Animals were injected with Ad-CMV or Ad-sFlt-1 and treated with MZe786 or MZe486 or aspirin. Results are expressed as mean (±SEM). Analysed by One-way ANOVA.

Supplementary Figure S2. Representative hematoxylin and eosin (HE) staining and labyrinth zone area of $Hmox1^{+/-}$ placenta showing marked reduction of the placental labyrinth zone (indicated by the dash lines) in Ad-sFlt-1 injected mice compared to Ad-CMV injected mice. Oral administration of MZe786 significantly reduced this structural alteration induced by overexpression of sFlt-1 in $Hmox1^{+/-}$ mice. Placenta of MZe486 and aspirin treated mice remain unchanged (scale bar represents 1mm).

Supplementary Figure S3. Pulsed wave Doppler images and representative sections of peak systolic (V_{max}) and minimum diastolic (V_{min}) of **(A)** uterine artery and **(B)** umbilical artery. Resistance index of **(C)** uterine artery and **(D)** umbilical artery of $Hmox1^{+/-}$ mice injected with Ad-CMV or Ad-sFlt-1 and treated with carrier, MZe786, MZe486 or aspirin. Resistance index was calculated using the following formula: $(V_{max} - V_{min})/V_{max}$.

Supplementary Figure S1

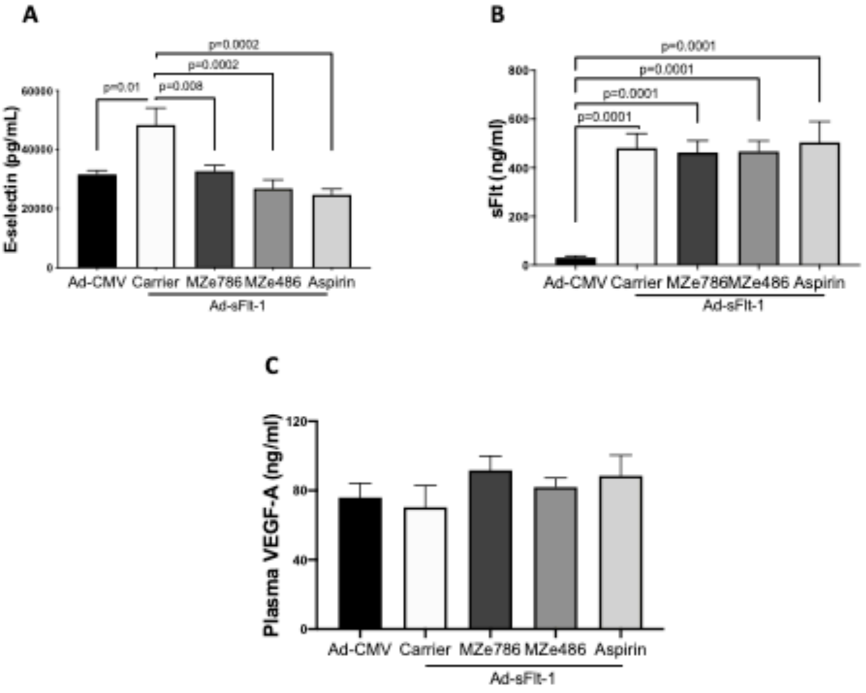

Supplementary Figure S2

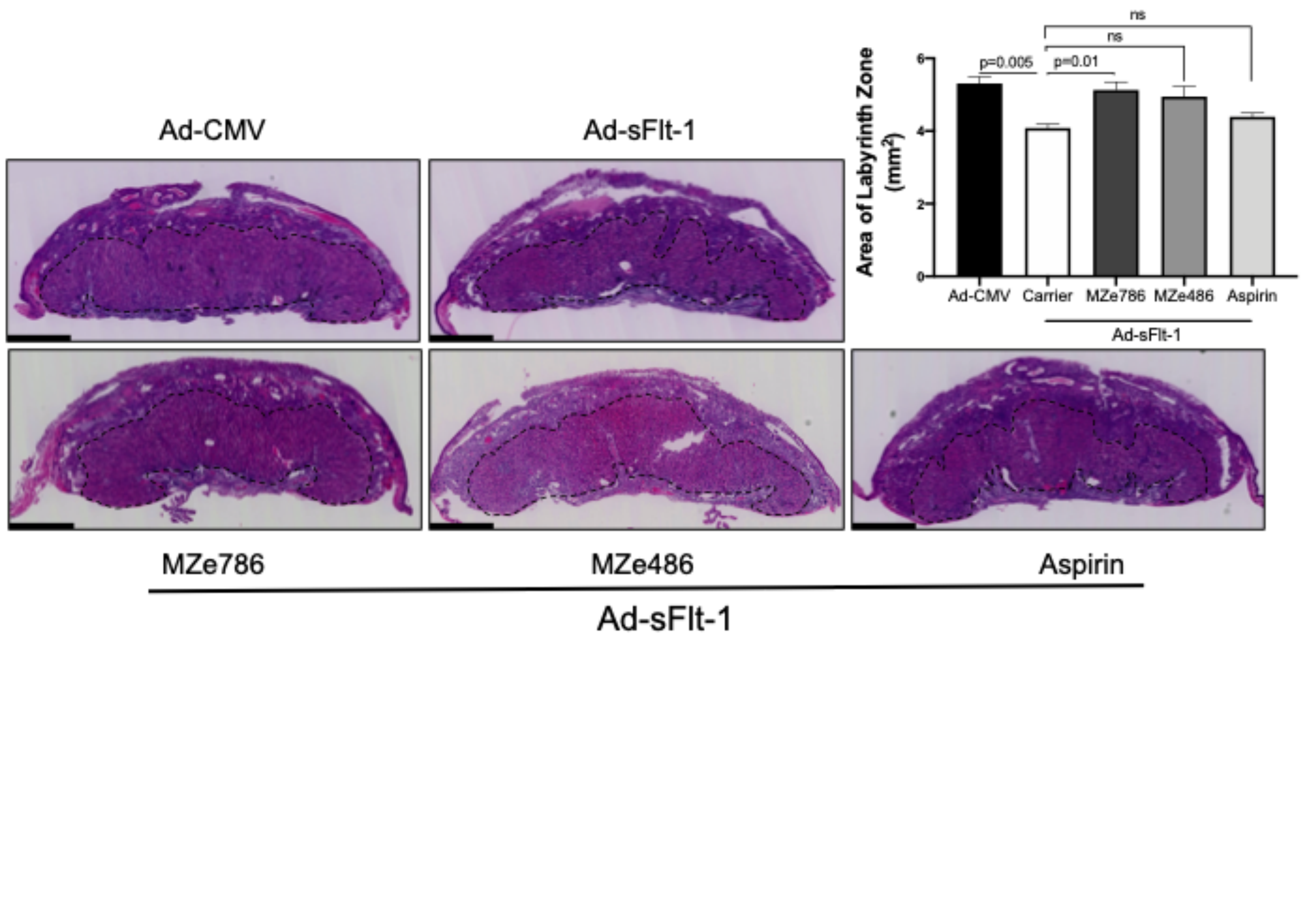

Supplementary Figure S3

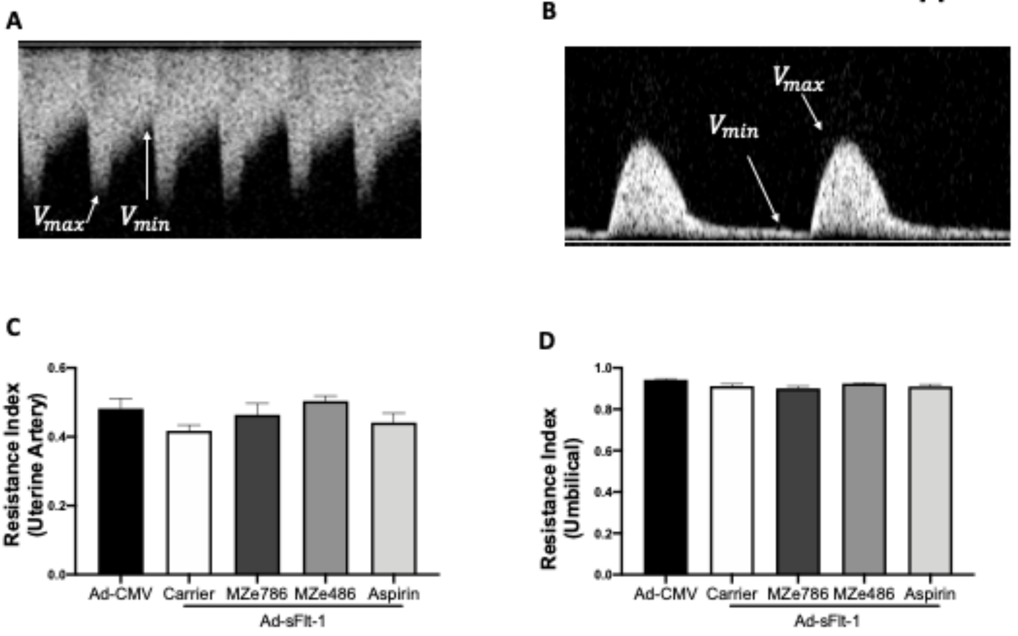

References

1. Sparatore A, Perrino E, Tazzari V, Giustarini D, Rossi R, Rossoni G, et al. Pharmacological profile of a novel H2S-releasing aspirin. Free Radical Biology and Medicine. 2009;46(5):586-92.
2. Wang K, Ahmad S, Cai M, Rennie J, Fujisawa T, Crispi F, et al. Dysregulation of hydrogen sulfide producing enzyme cystathionine gamma-lyase contributes to maternal hypertension and placental abnormalities in preeclampsia. Circulation. 2013;127(25):2514-22.
3. Sanchez-Aranguren LCR, H.; Ahmad, S.; Alzahrani, F.A.; Sparatore, A.; Wang, K.; Ahmed, A. MZe786 Rescues Cardiac Mitochondrial Activity in High sFlt-1 and Low HO-1 Environment. Antioxidants. 2020;9:598.
4. Hosoki R, Matsuki N, Kimura H. The possible role of hydrogen sulfide as an endogenous smooth muscle relaxant in synergy with nitric oxide. Biochem Biophys Res Commun. 1997;237(3):527-31.
5. Geng B, Chang L, Pan C, Qi Y, Zhao J, Pang Y, et al. Endogenous hydrogen sulfide regulation of myocardial injury induced by isoproterenol. Biochem Biophys Res Commun. 2004;318(3):756-63.
6. Lajin B, Francesconi KA. The hydrogen sulfide metabolite trimethylsulfonium is found in human urine. Sci Rep. 2016;6:27038.
